# Suspicion Distillation Gradient Descent Bit-Flipping Algorithm

**DOI:** 10.3390/e24040558

**Published:** 2022-04-15

**Authors:** Predrag Ivaniš, Srdjan Brkić, Bane Vasić

**Affiliations:** 1 School of Electrical Engineering, University of Belgrade, 11000 Belgrade, Serbia; srdjan.brkic@etf.rs; 2 Department of ECE, University of Arizona, Tucson, AZ 85721, USA; vasic@ece.arizona.edu

**Keywords:** bit-flipping algorithm, decoder re-initializations, gradient descent, iterative decoding, low-density parity-check codes

## Abstract

We propose a novel variant of the gradient descent bit-flipping (GDBF) algorithm for decoding low-density parity-check (LDPC) codes over the binary symmetric channel. The new bit-flipping rule is based on the reliability information passed from neighboring nodes in the corresponding Tanner graph. The name *SuspicionDistillation* reflects the main feature of the algorithm—that in every iteration, we assign a level of suspicion to each variable node about its current bit value. The level of suspicion of a variable node is used to decide whether the corresponding bit will be flipped. In addition, in each iteration, we determine the number of satisfied and unsatisfied checks that connect a suspicious node with other suspicious variable nodes. In this way, in the course of iteration, we “distill” such suspicious bits and flip them. The deterministic nature of the proposed algorithm results in a low-complexity implementation, as the bit-flipping rule can be obtained by modifying the original GDBF rule by using basic logic gates, and the modification is not applied in all decoding iterations. Furthermore, we present a more general framework based on deterministic re-initialization of the decoder input. The performance of the resulting algorithm is analyzed for the codes with various code lengths, and significant performance improvements are observed compared to the state-of-the-art hard-decision-decoding algorithms.

## 1. Introduction

Low-density parity-check (LDPC) codes [1] are powerful error correction codes that achieve performance near the Shannon limit with low decoding complexity [2]. These codes have been adopted in the fifth-generation standard for broadband cellular networks (5G NR) [3], digital video broadcasting standards for satellite communications (DVB-S2 and DVB-S2X, [4]), communication standards for Wi-Fi and WiMax networks [5,6], and passive optical networks [7].

The decoding algorithms of practical interest are typically based on a message-passing approach. The decoding procedures of LDPC codes are described by a bipartite graph—known as a Tanner graph—comprising two sets of nodes—variable nodes (VN) and check nodes (CN), and a set of edges between them. During decoding, messages are iteratively exchanged between VNs and CNs connected by an edge, i.e., the messages are sent to the neighbors. A prime example of a message-passing decoder is the belief propagation (BP) algorithm [1]. While the BP decoder exhibits excellent performance, this state-of-the-art algorithm requires complex computations in variable and check nodes. Consequently, its approximate variants, such as min-sum [8] and offset min-sum [9], are typically used in contemporary communication systems. All of these variants use “soft” (real-valued) messages and higher numerical precision of these messages to improve error correction capability at the cost of increasing hardware complexity and reducing throughput.

The BP-based decoding can be considered an iterative process of Bayesian inference and the messages are the representations of the corresponding probabilities. Although optimal for extremely long codewords, the performance of short and medium-length LDPC codes is far from optimal, due to increased error floors and the large number of decoding iterations needed to reach the desired decoding reliability. Given the fact that decoding latency is proportional to the codeword length, the aforementioned drawbacks limit the usefulness of LDPC codes in delay-sensitive applications, such as the URLLC (Ultra-Reliable and Low-Latency Communications) 5G radio service category [10]. Furthermore, the decoding complexity of message-passing algorithms increases significantly with the the number of edges that connects VNs and CNs. To mitigate the drawbacks of the BP decoder, finite alphabet iterative decoders (FAIDs) have been proposed in [11], and it is based on the idea to convey the local structure of the Tanner graph by employing a small number of additional bits in the messages.

On the other hand, bit flipping (BF) is the simplest iterative decoding algorithm that operates with one-bit messages and can provide very high throughputs when only hard decisions are available at the channel output. The BF algorithm has lower complexity when compared with the simplest hard-decision message-passing algorithm (Gallager-B), as there is no passing of the messages between the nodes in the bipartite graph (a comprehensive complexity analysis of the hard-decision decoders can be found in [12]). Although the implementation complexity of the BF algorithm is low, its performance is inferior when compared to the algorithms passing soft messages. The guaranteed error correction capability of the BF decoder is dominantly determined by two parameters of the bipartite graph: the girth and variable node degree (number of parity checks connected to the VN). As the girth grows logarithmically with the codeword length [13], the error correction capability of the BF-based decoders is poor for the short codes with small VN degrees.

Recent research attempts are aimed at providing decoding algorithms with a complexity comparable to the simple bit-flipping type of decoders but with a performance close to soft message-passing decoders. It has been shown that the gradient descent bit-flipping (GDBF) algorithm [14], together with the recently proposed improvements, has the potential to meet these requirements.

In this paper, we identify the drawbacks of the GDBF algorithm responsible for failing to correct some low-weight error patterns. We propose modifications that address these drawbacks, and thus improve the performance and convergence speed, even for codes with short blocklength and a small VN degree.

### 1.1. Related Work

The main idea of the GDBF algorithm [14] is that a non-linear objective function is defined for every VN in every iteration. It is calculated based on the received word from the channel, the current bit estimate for this VN, and the number of unsatisfied parity checks for that VN. Only the nodes with the maximum value of the objective function are “suspected” to be in error, and are flipped in that iteration. Although this approach results in faster convergence and increased correction capability when compared with typical bit-flipping-based algorithms [15,16], there are error patterns for which the decoding halts because a local minimum of the GDBF objective function is reached. One way to alleviate this drawback is to introduce randomness in VN or CN update functions. The algorithm that improves the decoder performance over the binary symmetric channel (BSC) by adding random binary sequences to the bit decisions during iterations is known as the probabilistic gradient descent bit-flipping (PGDBF) algorithm [17]. The impacts of randomness on performance improvement in the additive white Gaussian noise (AWGN) channel is analyzed in [18]. Another effect that adversely impacts convergence is the oscillatory behavior of a decoder—ns in a periodic fashion, and the algorithm loops endlessly. To avoid the “loops” in GDBF decoding, various approaches are proposed in the literature. An escaping scheme based on syndrome weight is proposed in [19]. In our work [20], we show that the same suspicious positions in subsequent iterations indicate the uncorrectable error patterns for the GDBF algorithm, known as *trapping sets*. In the same paper, we explained that the negative effect of the trapping sets can be avoided by applying multiple decoding attempts and random re-initialization (MUDRI). In fact, if the decoder is trapped in a local minima of the objective function, random perturbations can help the decoder to escape from these minima, and converge to a correct codeword. If the randomness is added to all VNs, the PGDBF decoder with restarts and re-initialization can approach the maximum likelihood (ML) performance after a sufficiently large number of iterations [21]. The multiple decoding with random re-initializations can also be applied to the other hard-decision decoders, and the corresponding theoretical analysis is given for the Gallager-B decoder in [22,23].

Speeding up the PGDBF decoder convergence has been studied by numerous research groups in recent years. One approach is to use the threshold that corresponds to the maximum value of the objective function from the previous iteration, as proposed in [24]. Another method is to modify the objective function to include the information about the number of flipping for every VN as in [25]. In the taboo-list random GDBF (TRGDBF) [26,27], a binary random sequence is added to the objective function, and VNs flipped in the previous iteration are prevented from flipping in the current iteration. If every VN has knowledge about the number of iterations since the last flip, this knowledge can also be used in the objective function to further improve the performance [28]. All these results indicate that a time dimension of every particular VN plays a significant role in the decision process, and this can be further optimized similarly as in the concept of finite alphabet iterative decoders (FAID) [11]. This observation is at the heart of our method too.

However, the algorithms that apply randomness have increased complexity, as pseudo random number generators, producing independent “random” sequences in every iteration, are required for every VN. It has been shown that hardware implementation of these algorithms is not a trivial task [27,29,30]; however, as shown in [31], deterministic improvements of the GDBF—typically based on adaptive thresholds—are effective for high-variable-degree codes only. The goal of this paper is to design a deterministic algorithm applicable to LDPC codes with low variable degree (less than five) with low implementation complexity and high convergence speed.

### 1.2. Summary and Organization

In this paper, we propose an algorithm that utilizes the reliability information passed from neighboring VNs. The name Suspicion Distillation Gradient Descent Bit-Flipping Algorithm (SDGDBF) reflects its main feature that in every iteration to every VN we assign a level of suspicion about its current bit value. How much we suspect a VN is not only used to decide whether a bit of this VN will be flipped, but also in each iteration, we determine the number of satisfied and unsatisfied checks that connect that VN with the other suspected VNs. In this way, in the course of iterations, we “distill” such suspicious bits and flip them. We show that information if the VN was flipped in the previous iterations can be combined with this variable-suspicion information to provide a significant performance improvement for various LDPC codes. Moreover, the algorithm is completely deterministic, i.e., it does not require randomness in any step during the decoding process.

The rest of the paper is organized as follows. In Section 2, the generalized GDBF algorithm for the BSC is presented, and an overview of the trapping sets is given. The SDGDBF algorithm is proposed in Section 3, where a few introductory examples are given, modification of the flipping rule is proposed, and the algorithm is formally defined. The complexity analysis is given in Section 4. Numerical results obtained by Monte Carlo simulations are presented in Section 5 for various LDPC codes. Optimization of the decoder parameters and further work is given in Section 6. The final conclusions are outlined in Section 7.

## 2. GDBF Decoding and the Impact of Trapping Sets

At the encoder side, a (*n*, *k*) binary LDPC code C of rate R=k/n is applied, where the information word with length *k* is represented with the corresponding codeword x with length *n*. The corresponding Tanner graph *G* consists of the set of variable nodes $V=\{v_{1},v_{2},\cdots ,v_{n}\}$ and the set of check nodes $C=\{c_{1},c_{2},\cdots ,c_{m}\}$. Parity check matrix *H* is the bi-adjacency matrix of *G*. Two nodes in *G* are neighbors if there is an edge between them, and the degree of a node is the number of its neighbors. In this paper, we consider regular LDPC codes where each variable node has degree γ and every check node has degree ρ. The sets of neighbors of nodes $v_{i}$ and $c_{j}$ are denoted as $N_{v_{i}}$ and $N_{c_{j}}$, respectively.

The transmission of a codeword x through the BSC with crossover probability $\alpha_{BSC}$ is modeled as a modulo-2 addition of a Bernoulli $B\;(1;\alpha_{BSC})$ random sequence of errors e, Pr($e_{i}$ = 1) = $\alpha_{BSC}$. The received word at the output of the BSC channel is then y=x⊕e, where ⊕ denotes logical XOR operation, i.e., bitwise addition modulo 2.

In the rest of this section, the decoder input will be denoted by r. At the start of the decoding, the input to the decoder is equal to the channel output (r=y). In a more general framework, where the overall decoding process can be divided into multiple stages, we allow the decoder input to be re-initialized to another value during the decoding (it will be further explained in the next section); therefore, the initial estimation ${\hat{x}}^{\left(0\right)}$ will be equal to a reference r in the analysis given below.

For the decoder input r, the decoder calculates the energy function for the *i*-th variable node in the *ℓ*-th iteration [14,17] as

(1) $$\Lambda_{i}^{\left(\ell \right)}\left({\hat{x}}^{\left(\ell \right)},r\right)={\hat{x}}_{i}^{\left(\ell \right)}⊕r_{i}+\sum_{c_{j}\in N_{v_{i}}}\underset{v_{k}\in N_{c_{j}}}{⨁}{\hat{x}}_{k}^{\left(\ell \right)},$$

where ⨁ denotes modulo-2 summation. Only those bits that have the maximum value of $\Lambda_{i}^{\left(\ell \right)}$ are called *suspicious nodes*, which means that they are most likely erroneous. Suspicious VNs are flipped in every iteration. The decoding is finished if all parity checks are satisfied or if the maximum number of decoding iterations, denoted by *L*, is elapsed.

**Figure 1:**
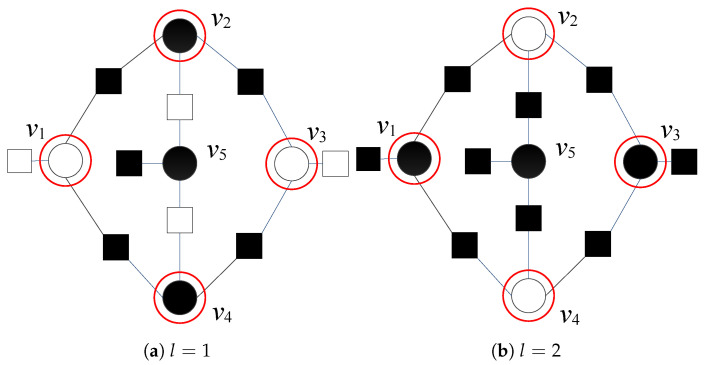
Decoding of three-bit error pattern by using GDBF decoder; flipping decisions after the first and the second iteration.

Even if the number of erroneous variables is low, they might be positioned in such a way that causes decoding oscillations and prevents convergence of the decoder to a valid codeword. This situation is illustrated in Figure 1, where correct variable nodes are represented with white circles 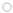, while erroneous variable nodes are represented with black circles 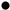. Satisfied parity checks are represented by white squares 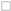, while unsatisfied parity checks are represented by using black squares 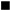. Variable nodes $v_{1}$, $v_{2}$, $v_{3}$, and $v_{4}$ are suspicious both in the first and second iterations (with $\Lambda_{i}^{\left(1\right)}\left({\hat{x}}^{\left(\ell \right)},r\right)=2$ and $\Lambda_{i}^{\left(2\right)}\left({\hat{x}}^{\left(\ell \right)},r\right)=4$, for i=1,2,3,4); therefore, the corresponding three-bit error pattern cannot be corrected.

In the PGDBF algorithm [17], the suspicious VNs are flipped with a predefined probability *p*. It is necessary to generate Bernoulli B(1;p) random variables $a_{i}^{\left(\ell \right)}$ (∀i,ℓ); only the variable nodes with $a_{i}^{\left(\ell \right)}=1$ and maximum value of $\Lambda_{i}^{\left(1\right)}\left({\hat{x}}^{\left(\ell \right)},r\right)$ are flipped. In the MUDRI algorithm, the decoding is performed in multiple stages, where a randomized received word is used as the decoder input in every particular decoding attempt.

Further improvement is proposed in the TRGDBF algorithm [27], where Bernoulli B(1;p) random variable $\lambda_{i}$ is added to the objective function, and taboo list $T_{i}$ of the VNs flipped in the previous iteration is created. If $T_{i}=1$ the corresponding node is prevented from flipping, and this can be obtained if we set $\Lambda_{i}{\left({\hat{x}}^{\left(\ell \right)},r\right)}^{\left(\ell \right)}=0$ for variable nodes on the tabu list. For the TRGDBF, the energy function can be written in the following form:

(2) $$\Lambda_{i}^{\left(\ell \right)}\left({\hat{x}}^{\left(\ell \right)},r\right)=\left({\hat{x}}_{i}^{\left(\ell \right)}⊕r_{i}+\sum_{c_{j}\in N_{v_{i}}}\underset{v_{k}\in N_{c_{j}}}{⨁}{\hat{x}}_{k}^{\left(\ell \right)}+\lambda_{i}\right)\left(1-T_{i}\right).$$

In a recent paper [28], the GDBF algorithm with momentum (GDBF-w/m) is proposed. The number of iterations from the previous flipping of $v_{i}$ is denoted by $w_{i}$, and the corresponding momentum vector is defined as $\mu =(\mu \left(1\right),\mu \left(2\right),\cdots ,\mu \left(w_{\max}\right))$, where we assume $\mu (w_{i})=0$ for $w_{i}>w_{\max}$. It has been shown in [28] that the convergence speed can be improved by using the correlation coefficient α. With the aim to easily provide integer values of energy functions, we introduce another coefficient β, as in the following expression

(3) $$\Lambda_{i}^{\left(\ell \right)}\left({\hat{x}}^{\left(\ell \right)},r\right)=\alpha \left({\hat{x}}_{i}^{\left(\ell \right)}⊕r_{i}\right)+\beta \left(\sum_{c_{j}\in N_{v_{i}}}\underset{v_{k}\in N_{c_{j}}}{⨁}{\hat{x}}_{k}^{\left(\ell \right)}\right)-\mu (w_{i}).$$

All of the above improvements of the GDBF algorithm can be summarized in Algorithm 1. The energy function is calculated according to the Equation (3). We set r=y in all algorithms except the MUDRI, where the decoder input r is re-initialized at every decoding attempt. For the GDBF, PGDBF, and MUDRI we set α=1,β=1,μ=0, and for the TRGDBF we set α=1,β=1,μ=γ+1. In the PGDBF, MUDRI, and TRGDBF algorithms, we set p<1 to incorporate randomness in the decoding process.
**Algorithm 1** Generalized GDBF Algorithm1: **Input:**
r,p,α,β,μ 2: $\forall v_{i}\in V:\;{\hat{x}}_{i}^{\left(0\right)}\leftarrow r_{i}$ 3: $s^{\left(0\right)}\leftarrow {\hat{x}}^{\left(0\right)}H^{T}$
$\left(\forall c_{j}\in C:\;s_{c}^{\left(0\right)}\leftarrow {⨁}_{j\in N_{j}}{\hat{x}}_{j}^{\left(0\right)}\right)$ 4: ℓ=0 5: **while**
$s^{\left(\ell \right)}\neq 0$ and l≤L
**do** 6:  ∀i∈V: Compute $\Lambda_{i}^{\left(\ell \right)}\left({\hat{x}}^{\left(\ell \right)},r\right))$ for given α,β,μ 7:  $\Lambda_{\max}^{\left(\ell \right)}\leftarrow \max_{i}\left(\Lambda_{i}^{\left(\ell \right)}\left({\hat{x}}^{\left(\ell \right)},r\right)\right))$ 8:  **for**
i←1 to *n*
**do** 9:   $a_{i}^{\left(\ell \right)}=B\left(1;p\right)$ 10:   **if**
$\Lambda_{i}^{\left(\ell \right)}\left({\hat{x}}^{\left(\ell \right)},r\right)=\Lambda_{\max}^{\left(\ell \right)}$
**then** 11:    ${\hat{x}}_{i}^{\left(\ell +1\right)}\leftarrow \left(1⊗a_{i}^{\left(\ell \right)}\right)⊕{\hat{x}}_{i}^{\left(\ell \right)}$ 12:   **else**
13:    ${\hat{x}}_{i}^{\left(\ell +1\right)}\leftarrow {\hat{x}}_{i}^{\left(\ell \right)}$ 14:   **end if**
15:  **end for**
16:  $s^{\left(\ell +1\right)}\leftarrow {\hat{x}}^{\left(\ell +1\right)}H^{T}$ 17:  l←l+1 18: **end while** 19: **Output:**
${\hat{x}}^{\left(\ell \right)}$


## 3. Suspicion Distillation GDBF Algorithm

Although the algorithms that introduce a randomness into the decoding process can be very effective, generating independent random sequences in all iterations increase complexity and there is a need for a deterministic algorithm with similar performances.

The suspicion distillation GDBF (SDGDBF) algorithm uses the existing GDBF algorithms as its components, and in some iterations invokes a procedure for locating satisfied parity checks connected to erroneous variable nodes and unsatisfied checks connected to correct variable nodes. This procedure, as well as the rule in which (a small fraction of) iterations are used to invoke it, is the main novelty in the paper. In those iterations when a modification is applied, we set μ=0. The intuition behind this is that the graph structure plays a more important role in these iterations than the messages in the previous iterations. In such an iteration, the reference is equal to the input estimate ${\hat{x}}^{\left(\ell \right)}$, and therefore ${\hat{x}}^{\left(\ell \right)}⊕r=0$. It is clear that the energy function is equal to the number of unsatisfied checks if β=1 (for any value of α). The output of the modification will be the updated reference r.

### 3.1. Modification of the Flipping Rule

The proposed modification follows the basic steps:

1. For the given codeword estimate ${\hat{x}}^{\left(\ell \right)}$, the set of candidates for flipping is expanded. The set of *suspicious* variable nodes $V_{S}^{\left(\ell \right)}$ contains VNs with $\Lambda_{i}^{\left(\ell \right)}\left({\hat{x}}^{\left(\ell \right)},{\hat{x}}^{\left(\ell \right)}\right)\geq \Lambda_{\max2}^{\left(\ell \right)}$. $\Lambda_{\max2}^{\left(\ell \right)}=\underset{i}{2\mathrm{ndmax}}\left(\Lambda_{i}^{\left(\ell \right)}\left({\hat{x}}^{\left(\ell \right)},{\hat{x}}^{\left(\ell \right)}\right)\right)$ denotes the second maximum value of the energy function, wherein the function 2ndmax(.) computes the second maximum value of its vector-valued argument. We set the indicator that the VN is suspicious $I_{S,i}^{\left(\ell \right)}=1$ if $v_{i}\in V_{S}$ (and equal to zero otherwise). The nodes with the energy function equal to $\Lambda_{\max}^{\left(\ell \right)}$ form a set of *very suspicious* variable nodes, denoted by $V_{VS}^{\left(\ell \right)}$;
2. For all VNs, we identify neighboring parity checks that are satisfied ($c_{j}=0$) and have at least one more suspicious VNs as their own neighbor. The number of such *unreliable satisfied* checks for the *i*-th variable node in the *ℓ*-th iteration is denoted as $N_{SC,i}^{\left(\ell \right)}$. The energy function is set to $\Lambda_{i}^{\left(\ell \right)}\left({\hat{x}}^{\left(\ell \right)},{\hat{x}}^{\left(\ell \right)}\right)=\gamma$ if the condition $N_{SC,i}^{\left(\ell \right)}+\Lambda_{i}^{\left(\ell \right)}\left({\hat{x}}^{\left(\ell \right)},{\hat{x}}^{\left(\ell \right)}\right)=\gamma$ is satisfied. In such a case, we add $v_{i}$ to the set $V_{S}^{\left(\ell \right)}$;
3. For $v_{i}\in V_{S}^{\left(\ell \right)}$, we update $N_{SC,i}^{\left(\ell \right)}$ and recalculate $\Lambda_{i}^{\left(\ell \right)}\left({\hat{x}}^{\left(\ell \right)},{\hat{x}}^{\left(\ell \right)}\right)\leftarrow \Lambda_{i}^{\left(\ell \right)}\left({\hat{x}}^{\left(\ell \right)},{\hat{x}}^{\left(\ell \right)}\right)+N_{SC,i}^{\left(\ell \right)}$. We update $\Lambda_{\max}^{\left(\ell \right)}$ and the corresponding set $V_{VS}^{\left(\ell \right)}$;
4. For $v_{i}\in V_{VS}^{\left(\ell \right)},$ we identify parity check neighbors that are not satisfied ($c_{j}=1$), and have at least one more very suspicious VN as their neighbor. The number of such *unreliable unsatisfied* checks for the *i*-th variable node in the *ℓ*-th iteration is denoted as $N_{NC,i}^{\left(\ell \right)}$, and number of all unsatisfied checks is denoted as $N_{UC,i}^{\left(\ell \right)}$. If $N_{NC,i}^{\left(\ell \right)}=N_{UC,i}^{\left(\ell \right)}$, we recalculate $\Lambda_{i}^{\left(\ell \right)}\left({\hat{x}}^{\left(\ell \right)},{\hat{x}}^{\left(\ell \right)}\right)\leftarrow \Lambda_{i}^{\left(\ell \right)}\left({\hat{x}}^{\left(\ell \right)},{\hat{x}}^{\left(\ell \right)}\right)-N_{NC,i}^{\left(\ell \right)}$, and update $V_{VS}^{\left(\ell \right)}$;
5. The node $v_{i}$ is flipped if $v_{i}\in V_{VS}$.

The modification is used in certain iterations to improve the reliability of the flipping decisions by updating the reference r, and it can be applied to any GDBF-based algorithm. In this section, we show a few illustrative examples of when the modification is applied to the standard GDBF algorithm. It can be noticed that, by applying the proposed modification, the decoder now searches for the parity checks that are misleading for the standard bit-flipping algorithm, i.e., for the parity checks that we call unreliable:

- Unreliable satisfied parity check, denoted by 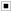, is satisfied but does not represent a reliable indicator that $v_{i}$ is correct;
- Unreliable unsatisfied parity check, denoted by 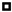, is unsatisfied but does not represent a reliable indicator that $v_{i}$ is erroneous;
- Reliable parity checks are those that do not belong to the above two unreliable categories, and are denoted in a typical fashion—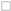 is a reliable satisfied check, while 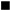 is a reliable unsatisfied check.

**Figure 2:**
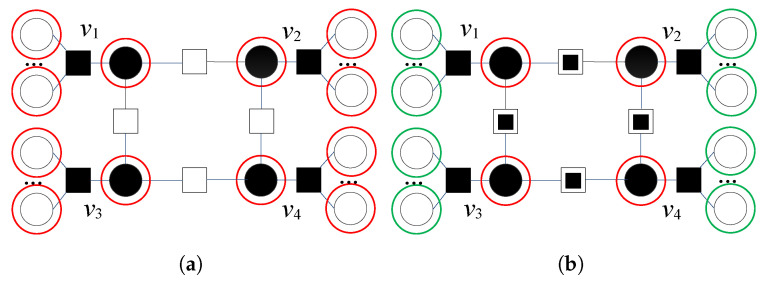
Decoding of four-bit error pattern with four very suspicious variable nodes, flipping decisions after the first iteration of GDBF, without modification and after the applied modification. Red circles correspond to very suspicious nodes; green circles to suspicious nodes. (**a**) without modification. (**b**) appliedmodification.

In Figure 2a, a typical four-bit error pattern for a code with girth g=8 and variable node degree γ=3 is presented. Variable nodes $v_{1}$, $v_{2}$, $v_{3}$, and $v_{4}$, as well as the other variable nodes connected to unsatisfied checks, are very suspicious both in the first and the second iteration (as $\Lambda_{\max2}^{\left(1\right)}=0$, we can notice that $V_{VS}^{\left(\ell \right)}=V_{S}^{\left(\ell \right)}$). Very suspicious VNs are marked with red circles. This four-bit error pattern is uncorrectable using the GDBF, as the GDBF decoder in all further iterations flips the same positions. We identify unreliable satisfied parity checks for every particular VN. Two unreliable satisfied checks (denoted by 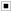) are neighbors of node $v_{1}$—one of them is connected to the suspicious node $v_{2}$, and the other is connected to the suspicious node $v_{3}$. A similar situation is found with the nodes $v_{2}$, $v_{3}$, and $v_{4}$; therefore, $N_{SC,i}^{\left(1\right)}=2$ and after the recalculation, we obtain $\Lambda_{i}^{\left(1\right)}\left({\hat{x}}^{\left(1\right)},{\hat{x}}^{\left(1\right)}\right)=3$, for i=1,2,3,4 (for the other VNs, the energy function is not updated). VNs that correspond to the updated set $V_{VS}$ are shown in Figure 2b, marked with red circles, while green circles correspond to the other VNs that belong to the updated set $V_{S}$. This four-error pattern is corrected in the same iteration when the modification is applied.

**Figure 3:**
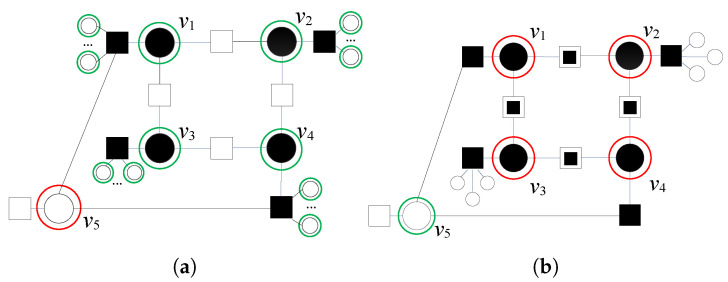
Decoding of four-bit error pattern with one very suspicious variable node and a large number of less suspicious-looking variable nodes, flipping decisions after the first iteration of the GDBF, without modification and after applying the modification. (**a**) Withoutmodification. (**b**) Appliedmodification.

The graph that corresponds to another four-bit error pattern is given in Figure 3a. Node $v_{5}$ has two unsatisfied parity checks, and only this node is very suspicious. In the next iteration, the energy function for $v_{5}$ is equal to $\Lambda_{5}^{\left(2\right)}\left({\hat{x}}^{\left(2\right)},r\right)=2$. As the same VN is suspicious in both iterations, this pattern cannot be corrected without a modification.

In this example, the only very suspicious node is $v_{5}$ (marked with a red circle); the other suspicious nodes are all VNs connected with one unsatisfied check (marked with green circles). There are $N_{SC,i}^{\left(1\right)}=2$ unreliable satisfied parity checks that connect $v_{1}$ with suspicious nodes $v_{2}$ and $v_{3}$. These satisfied checks are no more assumed as valid indicators if the node $v_{1}$ is correct, and the corresponding energy function is recalculated as $\Lambda_{1}^{\left(1\right)}\left({\hat{x}}^{\left(1\right)},{\hat{x}}^{\left(1\right)}\right)=3$. Repeating the same procedure for the other variable nodes, we easily obtain that the very suspicious nodes are now $v_{1}$, $v_{2}$, $v_{3}$, and $v_{4}$ (Figure 3b).

**Figure 4:**
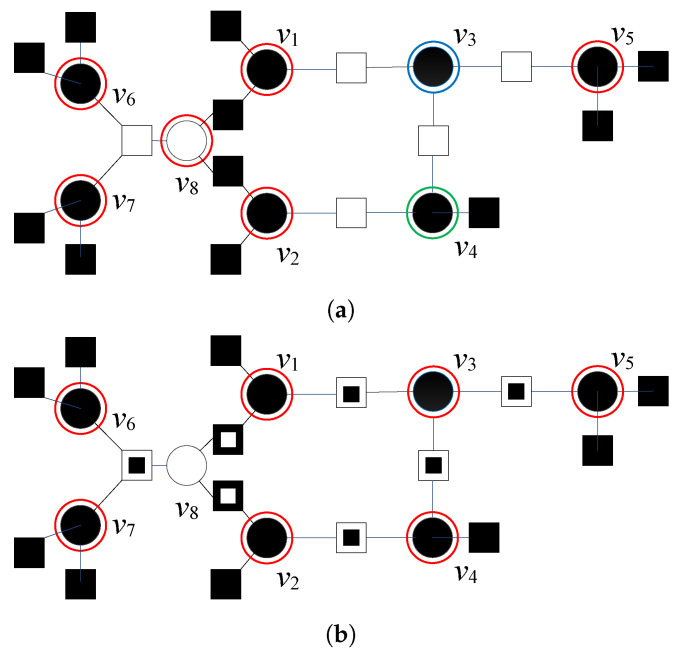
Decoding of seven-bit error pattern with four very suspicious variable nodes, after the first iteration of GDBF, without modification and after the applied modification. The blue circle corresponds to the node that should be added in the sets $V_{S}$ and $V_{VS}$. (**a**) Withoutmodification. (**b**) Appliedmodification.

In the following example, we analyze the correction of the seven-bit error pattern and illustrate a situation in which a node outside $V_{S}^{\left(l\right)}$ is a flipping candidate (Figure 4a).

Red circles denote six very suspicious nodes, with an energy function equal to $\Lambda_{\max}^{\left(1\right)}=2$, and green circle denotes another suspicious node $v_{4}$ with $\Lambda_{\max2}^{\left(1\right)}=1$. If we concentrate on $v_{3}$, we will see that it has all three satisfied parity checks as its neighbors. Each of these checks are connected to one suspicious VN. According to the proposed modification, these parity checks cannot be considered valid indicators of the correctness of $v_{3}$. These checks are satisfied but unreliable, and we obtain $N_{SC,3}^{\left(1\right)}=3$. Although $v_{3}$ is initially not a suspicious node ($\Lambda_{3}^{\left(1\right)}\left({\hat{x}}^{\left(1\right)},{\hat{x}}^{\left(1\right)}\right)=0$), it satisfies the condition $\Lambda_{3}^{\left(1\right)}\left({\hat{x}}^{\left(1\right)},{\hat{x}}^{\left(1\right)}\right)+N_{SC,3}^{\left(1\right)}=\gamma$, meaning that among the neighbors of the node $v_{3}$ there are not any reliable satisfied checks. Thus, we mark $v_{3}$ by a blue circle. Following the modification rule presented in step 2, we recalculate $\Lambda_{3}^{\left(1\right)}\left({\hat{x}}^{\left(1\right)},{\hat{x}}^{\left(1\right)}\right)=3$, and this node will be added in sets $V_{S}^{\left(\ell \right)}$ and $V_{VS}^{\left(\ell \right)}$.

According to step 3, the energy function values for the other suspicious nodes are also updated ($\Lambda_{i}^{\left(1\right)}\left({\hat{x}}^{\left(1\right)},{\hat{x}}^{\left(1\right)}\right)=3$, i=1,2,4,5,6,7,8). Notice that the parity check that connects $v_{2}$ and $v_{4}$ is also unreliable satisfied. The set $V_{VS}^{\left(\ell \right)}$ is updated.

Finally, node $v_{8}$ is connected with $N_{UC,8}^{\left(1\right)}=2$ unsatisfied checks. These parity checks are unreliable unsatisfied (denoted by 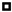 in Figure 4b), which have at least one more neighbor that is very suspicious, i.e., $N_{NC,8}^{\left(1\right)}=2$. As all unsatisfied checks are unreliable, we update $\Lambda_{8}^{\left(1\right)}\left(\hat{x},r\right)=1$. This node is removed from $V_{VS}$, and all errors in the pattern are corrected (see red circles in Figure 4b).

The exact formulation of the flipping rule modification is presented in Algorithm 2. The sets $V_{VS}$ and $V_{S}$ are initially created according to the value of the energy function, for $r=x^{\left(\ell \right)}$. Both sets are extended with the variable nodes $v_{i}$ that satisfy the condition defined in step 2, and the energy function of the suspicious nodes are updated following the rule from step 3. After the recalculation of the energy functions, we remove $v_{i}$ from $V_{VS}$ if it satisfies the condition from step 4. As explained later, the proposed modification is applied only in certain iterations.
**Algorithm 2** Modification of the flipping rule1: **Input:**
${\hat{x}}^{\left(\ell \right)}$ 2: $\forall v_{i}\in V$: Compute $\Lambda_{i}^{\left(\ell \right)}\left({\hat{x}}^{\left(\ell \right)},{\hat{x}}^{\left(\ell \right)}\right)$ for α=0,β=1andρ=0 3: $\Lambda_{\max}^{\left(\ell \right)}\leftarrow \max_{i}\left(\Lambda_{i}^{\left(\ell \right)}\left({\hat{x}}^{\left(\ell \right)},{\hat{x}}^{\left(\ell \right)}\right)\right),V_{VS}^{\left(\ell \right)}=\left\{v_{i}|\Lambda_{i}^{\left(\ell \right)}\left({\hat{x}}^{\left(\ell \right)},{\hat{x}}^{\left(\ell \right)}\right)=\Lambda_{\max}^{\left(\ell \right)}\right\}$ 4: $\Lambda_{\max2}^{\left(\ell \right)}\leftarrow \underset{i}{2\mathrm{ndmax}}\left(\Lambda_{i}^{\left(\ell \right)}\left({\hat{x}}^{\left(\ell \right)},{\hat{x}}^{\left(\ell \right)}\right)\right),V_{S}^{\left(\ell \right)}=\left\{v_{i}|\Lambda_{i}^{\left(\ell \right)}\left({\hat{x}}^{\left(\ell \right)},{\hat{x}}^{\left(\ell \right)}\right)\geq \Lambda_{\max2}^{\left(\ell \right)}\right\},I_{S,i}^{\left(\ell \right)}=0\;\forall i$ 5: **for**i←1 to *n*
**do** 6:  **if**
$v_{i}\in V_{S}^{\left(\ell \right)}$
**then** 7:   $I_{S,i}^{\left(\ell \right)}=1$ 8:  **end if**
9: **end for**
10: **for**i←1 to *n*
**do**
11:  $N_{SC,i}^{\left(\ell \right)}=\sum_{c_{j}=0\wedge c_{j}\in N_{v_{i}}}\vee_{v_{k}\in N_{c_{j}}/v_{i}}I_{S,k}^{\left(\ell \right)}$ 12:  **if**
$N_{SC,i}^{\left(\ell \right)}+\Lambda_{i}^{\left(\ell \right)}\left(\left({\hat{x}}^{\left(\ell \right)},{\hat{x}}^{\left(\ell \right)}\right)\right)=\gamma$
**then** 13:   $\Lambda_{i}^{\left(\ell \right)}\left({\hat{x}}^{\left(\ell \right)},{\hat{x}}^{\left(\ell \right)}\right)=\gamma ,V_{VS}^{\left(\ell \right)}=V_{VS}^{\left(\ell \right)}\cup v_{i},V_{S}^{\left(\ell \right)}=V_{S}^{\left(\ell \right)}\cup v_{i}$, $I_{S,i}^{\left(\ell \right)}=1$ 14:  **end if**
15: **end for**
16: **for**
i←1 to *n*
**do**
17:  **if**
$v_{i}\in V_{S}^{\left(\ell \right)}$
**then**
18:   $N_{SC,i}^{\left(\ell \right)}=\sum_{c_{j}=0\wedge c_{j}\in N_{v_{i}}}\vee_{v_{k}\in N_{c_{j}}/v_{i}}I_{S,k}^{\left(\ell \right)}$ 19:   $\Lambda_{i}^{\left(\ell \right)}\left(\left({\hat{x}}^{\left(\ell \right)},{\hat{x}}^{\left(\ell \right)}\right)\right)\leftarrow \Lambda_{i}^{\left(\ell \right)}\left(\left({\hat{x}}^{\left(\ell \right)},{\hat{x}}^{\left(\ell \right)}\right)\right)+N_{SC,i}^{\left(\ell \right)}$ 20:  **end if**
21: **end for**
22: $\Lambda_{\max}^{\left(\ell \right)}\leftarrow \max_{i}\left(\Lambda_{i}^{\left(\ell \right)}\left({\hat{x}}^{\left(\ell \right)},{\hat{x}}^{\left(\ell \right)}\right)\right)$, $V_{VS}^{\left(\ell \right)}=\left\{v_{i}|\Lambda_{i}^{\left(\ell \right)}\left({\hat{x}}^{\left(\ell \right)},{\hat{x}}^{\left(\ell \right)}\right)=\Lambda_{\max}^{\left(\ell \right)}\right\},I_{S,i}^{\left(\ell \right)}=0\;\forall i$,
23: **for**
i←1 to *n*
**do**
24:  **if**
$v_{i}\in V_{VS}^{\left(\ell \right)}$
**then**
25:   $I_{S,i}^{\left(\ell \right)}=1$
26:  **end if**
27: **end for**
28: **for**
i←1 to *n*
**do**
29:  **if**
$v_{i}\in V_{VS}^{\left(\ell \right)}$
**then**
30:   $N_{NC,i}^{\left(\ell \right)}=\sum_{c_{j}=1\wedge c_{j}\in N_{v_{i}}}\vee_{v_{k}\in N_{c_{j}}/v_{i}}I_{S,k}^{\left(\ell \right)}$
31:   $N_{UC,i}^{\left(\ell \right)}=\sum_{c_{j}\in N_{v_{i}}}{⨁}_{v_{k}\in N_{c_{j}}}{\hat{x}}_{k}^{\left(\ell \right)}$
32:   **if** ($N_{NC,i}^{\left(\ell \right)}>0)\wedge \left(N_{NC,i}^{\left(\ell \right)}=N_{UC,i}^{\left(\ell \right)}\right)$
**then**
33:    $\Lambda_{i}^{\left(\ell \right)}\left(\left({\hat{x}}^{\left(\ell \right)},{\hat{x}}^{\left(\ell \right)}\right)\right)\leftarrow \Lambda_{i}^{\left(\ell \right)}\left(\left({\hat{x}}^{\left(\ell \right)},{\hat{x}}^{\left(\ell \right)}\right)\right)-N_{NC,i}^{\left(\ell \right)}$
34:   **else**
35:    ${\hat{x_{i}}}^{\left(\ell \right)}=\hat{x_{i}}⊕1$
36:   **end if**
37:  **end if**
38: **end for**
39: **Output:**
$r={\hat{x}}^{\left(\ell \right)}$


Lines 1–9 in Algorithm 2 correspond to the first step defined on page 6. Lines 10–15 correspond to the second step, where we extend the set of suspicious VNs with the VNs that have no reliable satisfied parity checks as their neighbors. Lines 16–27 correspond to the third step, where we update the energy function for suspicious VNs, as well as the set $V_{VS}$. Lines 28–38 correspond to the fourth and the fifth step defined on page 6. The value of the energy function for very suspicious VNs is reduced if it has no reliable unsatisfied parity checks among its neighbors. If this condition is not satisfied, we flip the very suspicious variable node. Line 39 defines the output of the algorithm.

### 3.2. Modification Strategy and the Reference Update

In this paper, we consider the design of the deterministic low-complexity GDBF-based algorithm with superior performance. To avoid an increase in complexity, it is desirable not to apply the previously proposed modifications in every iteration of the decoding process. In fact, the best performance is obtained if the modification is applied in the following cases: (a) only for the patterns that cannot be decoded by using the basic algorithm and (b) in the iterations when it is detected that the decoding algorithm does not converge.

If the GDBF based on Equation (1) is used as a basic algorithm, and the same positions are selected for flipping in two subsequent iterations (example from Figure 1), it is obvious that the decoding will be terminated without any success if the modification is not applied. To implement the corresponding “alarm”, it is necessary to remember the flipping positions in at least two subsequent iterations.

If we use the GDBF-w/m as a basic algorithm, the trapping sets with short cycles will be corrected, and there is no need to check if all flipping positions are repeated after a certain number of iterations. After a certain number of iterations, even for this type of decoder, the probability of correcting any error pattern is negligible (this is illustrated in Section 5). This number of iterations after which no more bits are corrected is estimated empirically and denoted by $K_{1}$. $K_{1}$ determines the right moment to apply the modification described in Section 3.1.

**Figure 5:**
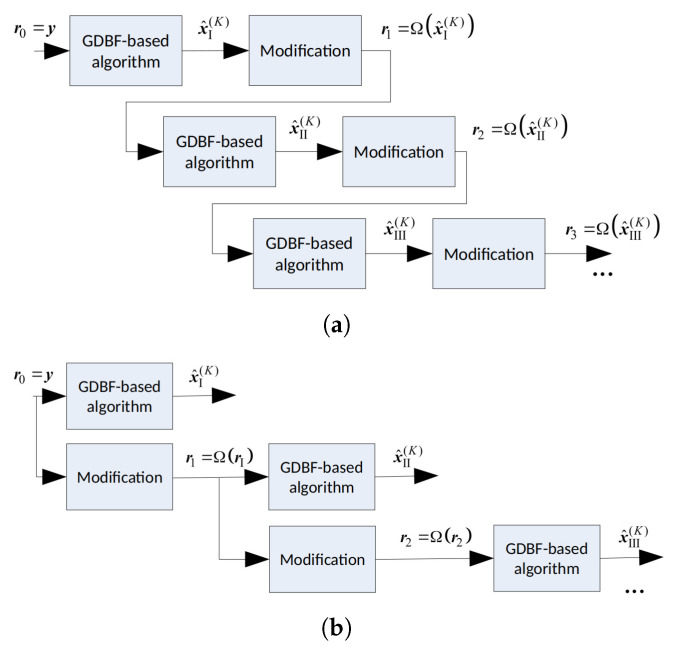
Reference updating after the decoding rounds (*K* iterations each); two approaches. (**a**) Modificationsappliedontheestimates. (**b**) Successivemodificationsappliedonthereceivedword.

The codeword estimate obtained after the applied modification can be used as a new reference r; therefore, the modification formulated in Algorithm 2 can be considered as the function Ω(.) that converts a codeword estimate ${\hat{x}}^{\left(\ell \right)}$ into the new reference r, that will be used as the input of the basic decoder (with any GDBF-based algorithm) in the next decoding round, during the next $K_{2}$ iterations. Various decoding strategies are possible. Modification can be applied to the codeword estimate obtained after the first attempt of $K_{1}$ iterations of the GDBF-based algorithm, denoted by ${\hat{x}}_{I}^{\left(K_{1}\right)}$. In this scenario, the modification should be applied after every decoding attempt, as illustrated in Figure 5a; however, this is not useful if the Hamming distance between the codeword and its estimate is too large. The other approach would be to apply successive modifications to the received word, and use the corresponding vectors as the references in multiple decoding attempts (Figure 5b). We focus on this approach in the next section.

### 3.3. The Decoder Input Re-Initializations

In our previous work, we noticed that the random re-initializations of the decoder input combined with the multiple decoding attempts resulted in more significant performance improvement [20].

**Figure 6:**
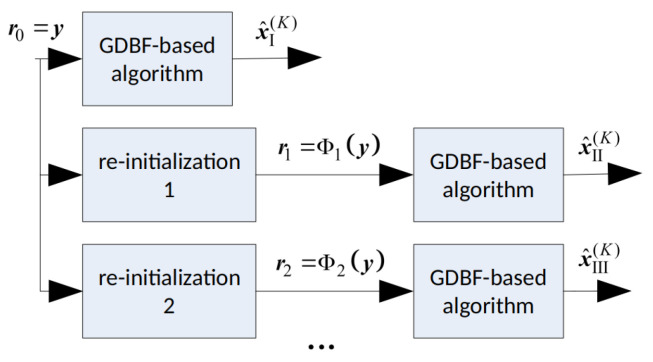
Reference updates by using the deterministic re-initializations; illustration of the multiple decoding attempts.

**Figure 7:**
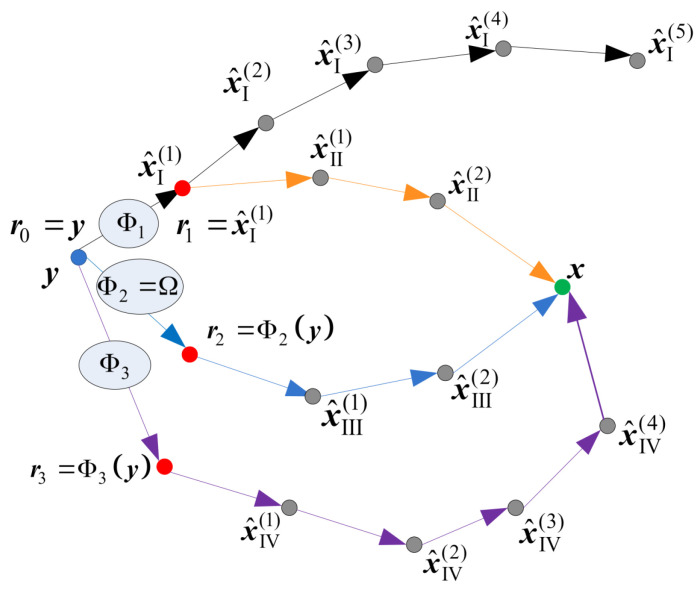
Various strategies for the decoder input re-initializations.

In this paper, we apply a deterministic re-initialization, where the reference update can be performed by using a deterministic function $\Phi_{q}\left(.\right)$ that transforms the received word y into the reference $r_{q}$. This reference will further be used as the initial estimate in the corresponding decoding attempt, as illustrated in Figure 6. The decoding trajectories are given in Figure 7. The re-initializations are applied only if the basic GDBF decoder fails to decode the codeword after $K_{1}$ iterations (decoding trajectory represented with black lines). Red dots represent the updated references that should be used as the input of the basic GDBF decoder in the corresponding re-initialization, and arrows without additional notations correspond to one iteration of the basic GDBF decoder, described in Algorithm 1.

The following approaches, illustrated in Figure 7, have been shown to be very effective:

- The codeword estimate after the first iteration is used as a new input for the decoder, i.e., flipping decisions in the first iteration define how to obtain the reference from y (the decoding trajectory represented by an orange color in Figure 7), and the function $\Phi_{1}\left(.\right)$ that assigns y to reference $r_{1}$ corresponds to one iteration of the GDBF decoding algorithm described in Algorithm 1;
- The modification described in Algorithm 2 can also be considered as a special case of the reference re-initialization if we apply transformation $r_{2}=\Phi_{2}\left(y\right)=\Omega \left(y\right)$ (the decoding trajectory represented by the blue color in Figure 7);
- The reference $r_{3}$ used in the purple trajectory represents the received sequence y changed in a single bit position. The position of the received bit that will be changed (flipped) is chosen among bits that were flipped during the first few iterations of the basic algorithm run prior to the modification.

**Figure 8:**
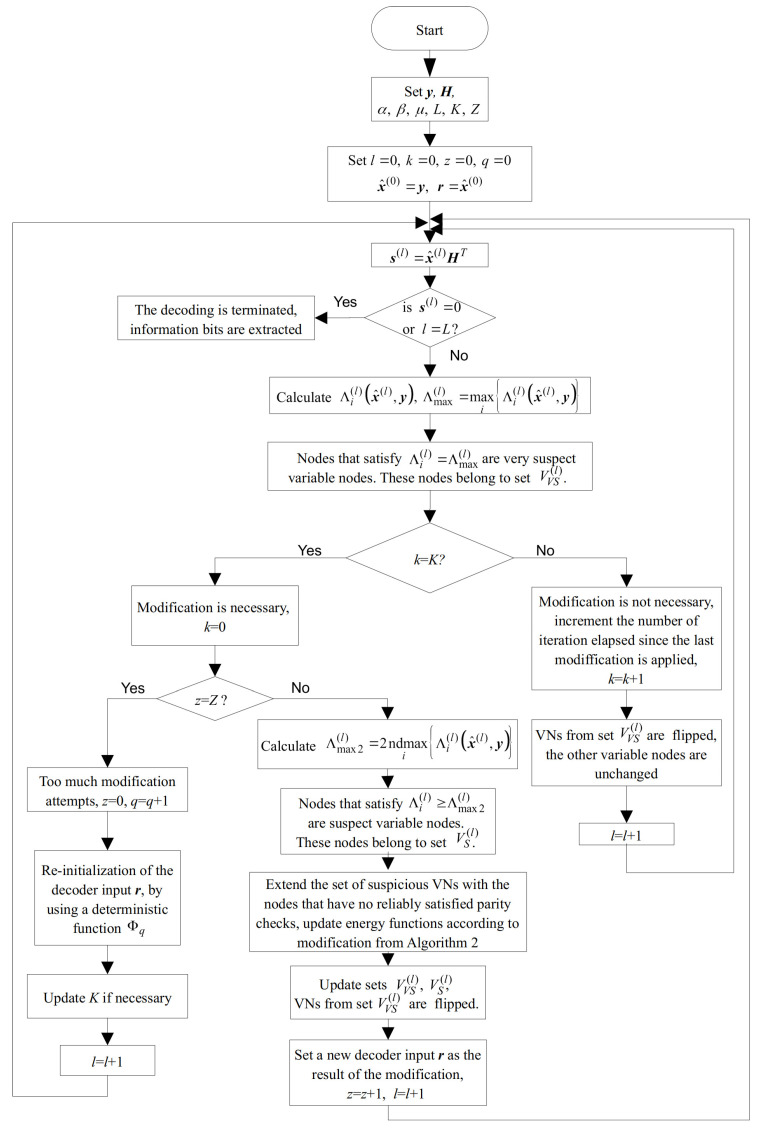
The flowchart of the SDGDBF algorithm.

Finally, the overall flowchart of the SDGDBF algorithm is presented in Figure 8. It is based on Algorithm 1, where the energy function is calculated by Equation (3). After *K* iterations, modification defined in Algorithm 2 is applied in one iteration, and the reference is updated. If the modification is repeated *Z* times, the re-initialization is applied. The decoding stops if a codeword is reached, or if a maximum number of iterations is elapsed. As we show in the next section, even if parameters *K* and *Z* are determined empirically, a significant performance improvement compared to the existing GDBF-based algorithms is possible.

## 4. Implementation Complexity

### 4.1. Computational Complexity

In this subsection, we examine the complexity of the SDGDBF decoder and make comparisons with the state-of-the-art GDBF and PGDBF decoders. Without going into a specific hardware realization, we estimate the number of arithmetical and logical operations required to perform a single decoding iteration. According to Algorithm 1, there are four types of operations in the GDBF-w/m decoder: binary XOR operations with ρ inputs, integer additions, a global maximization (with *n* inputs), and integer comparisons (Table 1). The global maximization is implemented by n−1 integer comparisons, and it was estimated that the GDBF has 40% larger complexity when compared to the BF algorithm [12]. The GDBF-w/m decoder has *n* extra integer additions (with the momentum vector).

The modification, proposed in Algorithm 2, contains all operations of the GDBF-w/m decoder; however, it also requires computational resources to perform: (i) integer additions, which are proportional to sizes of sets $V_{S}$ and $V_{VS}$ (both are lower than *n*); (ii) two additional global maximization operations as well as additional integer comparators; (iii) binary OR operations and single bit comparators. Given the fact that the complexity of the modification varies depending on the decoded sequence, in Table 1 we give an upper complexity bound. It should be noted that in the complexity analysis, we neglect possible integer multiplications, required for α and β scaling, given the fact that α and β are usually small integers (the most often values are α=β=1), and that such scaling can be implemented through decimal point shifting, or small lookup tables. We also do not include logical circuits for syndrome calculation, nor do we include circuits that count iterations from previous variables flips, needed for the momentum term calculations. All the neglected operations are common to the GDBF-w/m and the proposed modification, and do not significantly influence the relative complexity ratio between the two solutions.

Roughly, an iteration where the proposed modification is applied is three times more complex than one iteration of the GDBF-w/m decoder; however, we use the modification only in cases that the GDBF-w/m fails to decode the received sequence in $K_{1}$ iterations, meaning that Algorithm 2 is rarely used. For example, when the BSC crossover probability is equal to $\alpha_{\mathrm{BSC}}=0.01$ and average number of iterations is equal to $I_{av}=1.87$. In this case, the GDBF-w/m decoder applied on Tanner code for $K_{1}=25$ iterations lowers the FER to approximately $10^{-5}$, meaning that the modification is employed, on average, only once in $10^{5}$ decoded sequences. In the rest of the decoding process, the modification is periodically applied every $K_{2}=20$ iterations and the computational complexity at the worst case can be estimated as $C_{SDGDBF}=\left(19/20\right)\times \left(C_{GDBFwm}\right)+\left(1/20\right)\times \left(3\times C_{GDBFwm}\right)=1.1\times C_{GDBFwm}$, where $C_{GDBFwm}$ denotes computational complexity of the GDBF-w/m decoder.

On the other hand, employing random generators in the PGDBF decoder through linear feedback shift registers, as the alternative to the approach proposed in this paper, leads to a nine times larger memory requirements and close to 60% computational increase, compared to the GDBF decoder [32]. The random generators are used in every iteration, which means that they influence evenly the worst case and average complexities. It should be emphasized that a less complex random generator architecture is proposed in [29]; however, it is uncertain how it will influence the performance of arbitrary chosen code.

### 4.2. Efficient Implementation

**Figure 9:**
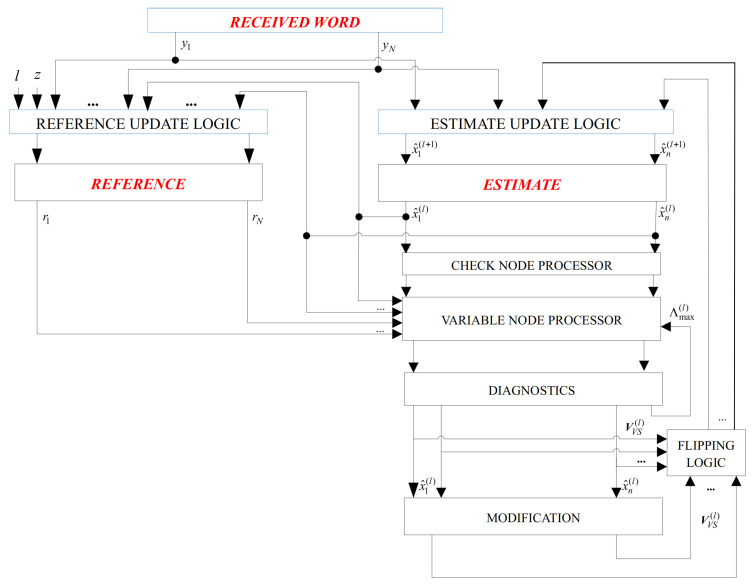
Main blocks in the implementation of the SDGDBF algorithm.

Figure 9 illustrates the basic implementation concept of the proposed algorithm. During the decoding, we use three *n*-bit memory registers:

- **RECEIVED WORD** contains the received word from the channel, and it is stored here for possible re-initializations.
- **REFERENCE** is used to store vector r, which will be used as the decoder input in the current attempt. Initially, this register contains the received word from the channel (r=y), and its update is very simple. If re-initialization is applied, the reference is equal to a slightly changed received word, as explained in the previous section.
- **ESTIMATE** is the memory that contains the current estimation of the codeword, i.e., the estimation after the *ℓ*-th iteration, with initial value ${\hat{x}}^{\left(0\right)}=y$. In addition, the information if the node is suspicious or very suspicious is stored here.

Parity checks for the estimated codeword are realized using XOR gates with ρ inputs. A variable node processor is realized using majority logic (MAJ) gates. **DIAGNOSTICS** uses counters to determine parameters l,k,z and logic gates that compare it with predefined values L,K,Z, respectively (to check if a modification is necessary). Further, it calculates the thresholds for MAJ gates in the *ℓ*-th iteration.

When compared to the GDBF-w/m algorithm, we have an additional *n*-bit memory register **REFERENCE** (in the GDBF, received word y is used as the reference in all iterations), memory for storing suspicious, and very suspicious positions. Block **MODIFICATION** is used for modifying the flipping rule (that is not used in all iterations). This block uses one OR logic gate with ρ−1 inputs in each check node, as well as the integer adders that calculate $N_{SC,i}^{\left(\ell \right)}$ and $N_{NC,i}^{\left(\ell \right)}$; however, only *m* OR gates are required, as the 3m OR operations in steps 2–4 are consequently performed on the same logical gates. The same maximization circuit that is used to find the maximum of the energy functions in the GDBF-w/m algorithm can be also used to find the second maximum, and to update the maximum of the energy function in step 3. In addition, the same integer comparators and integer adders that are used in step 1 can be reused to perform integer comparisons in further steps.

## 5. Numerical Results

In this section, we illustrate the decoding performance of the SDGDBF decoder. It is compared with the decoding performance of the existing GDBF-based algorithms. In addition, the floating-point implementation performance of the sum-product algorithm (SPA) is included for reference. To make a fair comparison, the maximum number of iterations is set to L=300 for all bit-flipping-based algorithms and L=50 for the SPA algorithm, if not specified otherwise. The same assumption is used in the previous relevant papers [27,28], and it is based on the throughputs achieved in hardware implementations [33]. The numerical results are obtained by using Monte Carlo simulations, and the frame error rate (FER) estimation is terminated when at least 100 failed codewords are collected.

In all performed simulations, the GDBF algorithm with momentum is selected as a base for the SDGDBF decoder (the energy function is calculated by using expression Equation (3)). Although the modifications presented in Section 3 can also be applied to the other types of the decoders, we have selected the GDBF-w/m to reduce the decoder complexity as there is no need to apply a random generator and there is no need to detect the occurrence of uncorrectable error patterns with short cycles in this case.

We first compare the FER performance of the proposed algorithm with the other GDBF-based algorithms for the (155,64) Tanner code, with code rate R=0.4. The code has a quasi-cyclic structure with construction proposed in papers [34,35] and it can be represented by using bipartite graph with n=155 variable nodes and m=93 check nodes, with VN degree γ=3, CN degree ρ=5, and girth g=8.

**Figure 10:**
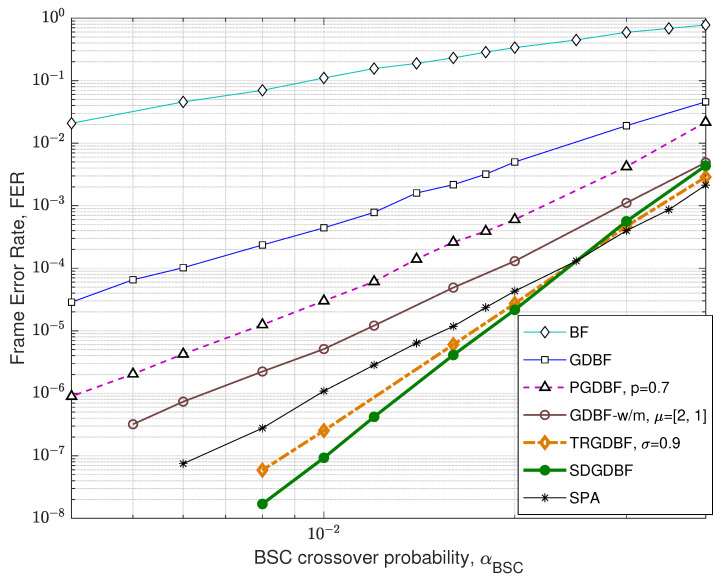
Performance of the various decoding algorithms, Tanner code (n=155, γ=3, ρ=5).

The FER performance for the Tanner code is presented in Figure 10. For the GDBF-w/m and the SDGDBF, we set weighting factors α=2, β=2, and momentum vector μ=[2,1]. In the SDGDBF algorithm, the modification described in Algorithm 2 is applied after K=25 iterations. Further periodical re-initializations, based on the flipping of only one suspicious position in the received word (as described in Section 3.3), are applied on every K=10 iterations and followed by Z=1 modification each. This strategy resulted in superior performance in the error floor region and the SDGDBF with L=300 iterations overcomes the performance of the floating-point SPA with L=50 if $\alpha_{BSC}<0.025$.

**Figure 11:**
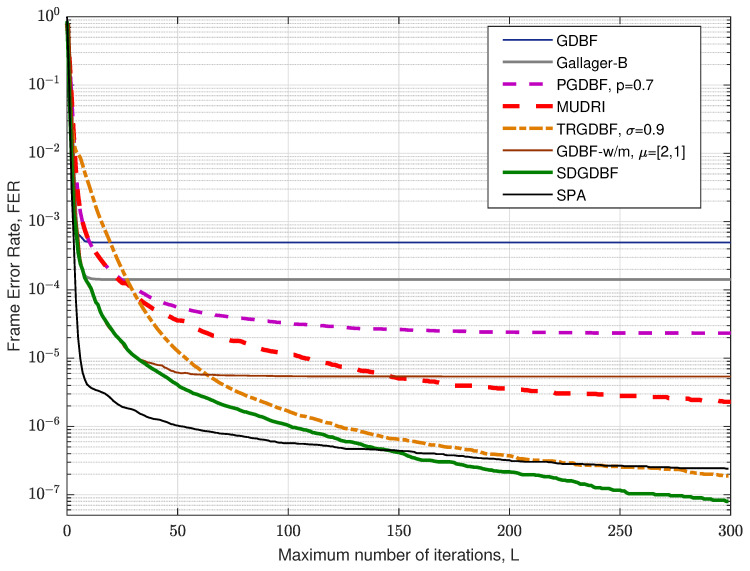
Convergence speed of the various decoding algorithms, Tanner code.

The FER performance for the Tanner code for various values of *L* is presented in Figure 11, for crossover probability $\alpha_{BSC}=0.01$. It is clearly visible that the deterministic hard-decision algorithms (GDBF, Gallager-B, and GDBF-w/m) do not improve performance after a certain number of iterations. The GDBF saturates after ten iterations, and further iterations cannot reduce the FER level anymore. The GDBF-w/m algorithm corrects most of the correctable error patterns in the first 20–30 iterations and saturates after 50 iterations; therefore, it is a wise strategy to apply the modification when the most of the correctable patterns are corrected by using the base algorithm. We empirically set the initial value $K_{1}=25$ in the case when the GDBF-w/m is chosen as a base algorithm. This value can be further reduced to $K_{2}=10$ after re-initializations are applied, as the benefit of frequent re-initialization is estimated to be higher than the benefit of a larger parameter *K*. It can be noticed that the SDGDBF algorithm has superior performance when compared with the MUDRI algorithm with random re-initializations on $L_{1}$ iterations, and the TRGDBF algorithm that combines random sequences with the tabu-list principle. In addition, it is visible that the SDGDBF algorithm with L=100 has the same performance as the SPA algorithm with L=50. If L>150, the SDGDBF algorithm results in better performance than the SPA algorithm for the same value of *L*.

**Figure 12:**
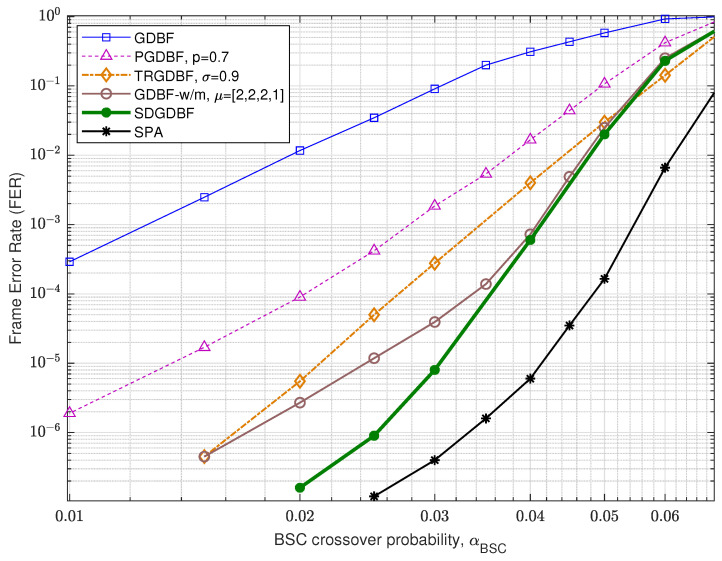
Performance of the various decoding algorithms, QC-LDPC code (n=1296, γ=3, ρ=6).

**Figure 13:**
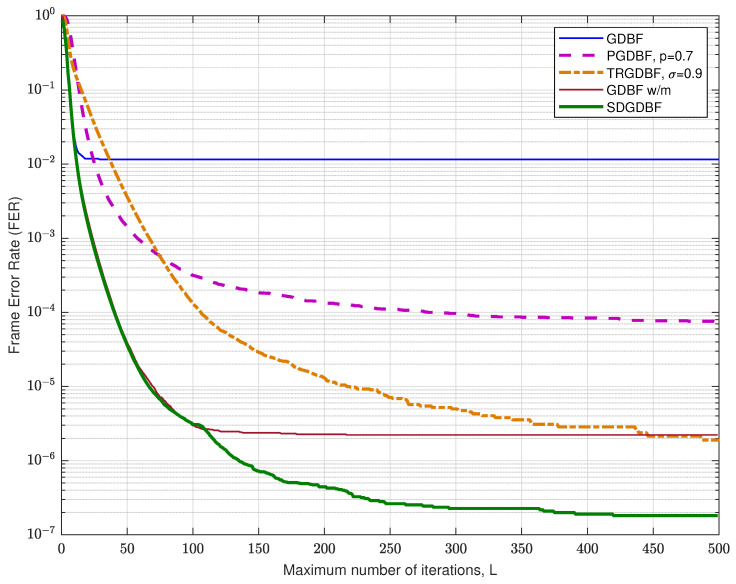
Convergence speed of the various decoding algorithms, QC-LDPC code with n=1296.

Performance of the analyzed GDBF-based algorithms for (1296, 648) QC-LDPC code with code rate R=0.5 is presented in Figure 12. The corresponding construction method is presented in paper [33], and the code can be represented by using a bipartite graph with n=1296 variable nodes and m=648 parity checks, with VN degree γ=3, CN degree, ρ=6, and girth g=8. For this code, it is obvious that the TRGDBF and the GDBF-w/m algorithms have much better performance when compared to the GDBF and the PGDBF algorithms. This illustrates the importance of the momentum for the longer codes. If the modification proposed in Algorithm 2 is applied to the GDBF-w/m, further performance improvement is possible if we choose the appropriate decoding parameters. The selection of the decoder parameters are explained with the help of the results presented in Figure 13 (given for $\alpha_{BSC}=0.02$). The decoding convergence for the GDBF-w/m is much faster compared to the TRGDBF algorithm, and the GDBF-w/m provides lower FER values when compared to the GDBF and the PGDBF for any value of *L*.

Based on its fast convergence, the GDBF-w/m is selected as an optimal candidate for the basic algorithm. The corresponding weighting factors α=1, β=2 and momentum vector μ=[2,2,2,1] are taken from paper [28] (the weighting factors are adjusted to uni-polar codewords, when compared to bi-polar implementation presented in [28], Table I).

As the GDBF-w/m algorithm corrects almost all correctable error patterns in the first 150 iterations, we empirically set the initial value $K_{1}=110$. This value can be reduced to $K_{2}=50$ if re-initializations are applied to boost the benefit of frequent re-initialization. Every re-initialization is followed with Z=1 modification, so there are approximately 100 iterations in every re-initialization cycle. It can be noticed that the optimal value of *K* is generally sensitive to BSC crossover probability. For short codes, such as (155, 64) Tanner code, one value of *K* can be used for a wide range of $\alpha_{BSC}$ without significant performance degradation; however, for longer LDPC codes, it is highly desirable to determine parameter *K* for any value of $\alpha_{BSC}$.

**Figure 14:**
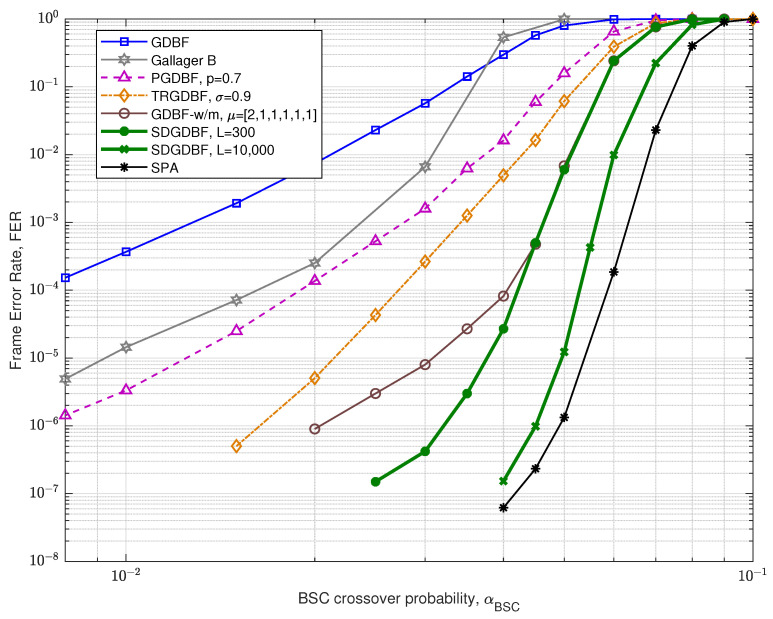
Performance of the various decoding algorithms, Margulis code (n=2640, γ=3, ρ=6).

Finally, we consider (2640, 1320) Margulis code with the code rate R=0.5 [36], which can be represented by using bipartite graph with n=2640 variable nodes and m=1320 parity checks, with VN degree γ=3, CN degree ρ=6, and girth g=8. The corresponding FER performance is presented in Figure 14. It is obvious that the SDGDBF algorithm improves the performance of the GDBF-w/m algorithm (with parameters α=1, β=2 and μ=[2,1,1,1,1,1]) in the error floor region even for a small value of *L*. If parameter *L* can be further increased, the performance is comparable with the floating point decoding algorithms based on the message-passing principle.

**Figure 15:**
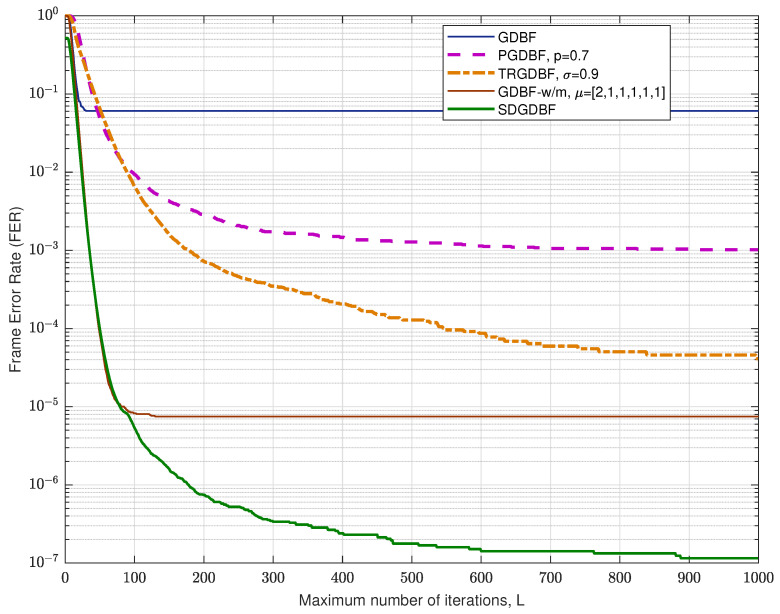
Convergence speed of the various decoding algorithms, Margulis code.

The performance of the analyzed GDBF-based algorithms for $\alpha_{BSC}=0.03$ and various values of *L* is presented in Figure 15. It is obvious that the GDBF algorithm with momentum has good performance even for a limited number of decoding iterations. If the modifications presented in Section 3 are applied for $K_{1}=80$, Z=1, the resulting SDGDBF algorithm has the potential to reach the performance of the SPA algorithm after a large number of iterations.

## 6. Optimization of the Decoder Parameters and Further Work

**Figure 16:**
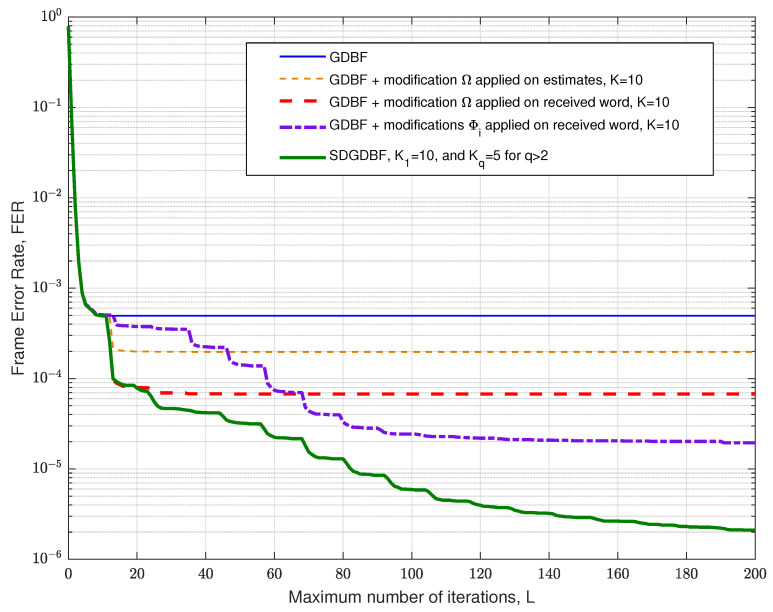
Various strategies for re-initializations, classical GDBF as the base algorithm, Tanner code.

It has been shown that various deterministic functions can be applied for the re-initialization of the decoder input in multiple decoding attempts. In Figure 16, we present the numerical results that correspond to various decoding strategies, and the results are given for the short Tanner code and $\alpha_{BSC}=0.01$. The FER plot is given for the classical GDBF algorithm for the BSC channel, i.e., the effect of momentum is not considered in this figure.

In the future work, we will consider the following intriguing directions related to the decoding strategies presented in Section 3.2 and Section 3.3:

- The modification described in Algorithm 2 is applied on a codeword estimate after K=10 iterations, i.e., $r=\Omega ({\hat{x}}^{\left(10\right)})$, and the procedure is repeated as illustrated in Figure 5a. In this case, the performance improvement is minor, as the Hamming distance between the estimate and the codeword is usually too large after so many iterations. Furthermore, this approach can result in miscorrection (the estimate is equal to another codeword);
- The modification described in Algorithm 2 is applied to the received word, i.e., r=Ω(y), and the procedure is repeated as illustrated in Figure 5b. This approach results in a more significant improvement, and miscorrections are avoided. As in the first approach, the FER improvement is most significant for the first modification and diminishes when it is repeated (for a longer code, further improvement can be eventually visible for larger *Z*);
- On the other hand, we can modify the algorithm to remember a few positions of the flipped bits during the first few iterations of the GDBF algorithm, denoted by $i_{1},i_{2},i_{3},\cdots$. In the *q*-th decoding attempt, the reference is obtained as $r_{i_{q}}=y_{i_{q}}⊕1$ and $r_{i_{j}}=y_{i_{j}},j\neq q$. The corresponding transformation $r=\Phi_{q}\left(y\right)$ results in the flipping of only one bit in the received word. This approach prevents saturating of the FER, as illustrated in Figure 16.

The previously described approaches can be combined in successive decoding attempts for a maximum number of iterations per attempt denoted by $K_{q}$. We estimated that the largest performance improvement can be obtained if the proposed modification (given by transformation Ω(.)) is followed by re-initialization based on $\Phi_{q}\left(.\right),\forall q$ (as proposed in the flowchart given in Figure 8). Empirically, we estimate that the GDBF decoder should be initially run for $K_{1}=10$ iterations, and we set $K_{q}=5$ for all re-initializations.

In our future research, we will try to further optimize parameters $K_{q}$ under the condition ($K_{1}+K_{2}+\cdots =L$), as well as the sequence of applied deterministic functions. In addition, we will concentrate on the extension of the proposed method to the irregular codes, where the variable and check node degrees are node dependent. In such a case, certain conditions (such as in step 2, defined on page 6) will depend on the variable node degree, and therefore the scaling coefficients α and β should be adapted for various variable nodes. We believe that the optimization of these parameters for a specific code by using machine learning techniques will result in the additional improvement of the decoder performance.

## 7. Discussion and Conclusions

By combining the concept of momentum and the graph properties of the typical trapping sets, we have proposed a deterministic modification of the GDBF algorithm. In previously proposed GDBF-based algorithms, the flipping decisions were based on the energy function that takes into account the number of unsatisfied parity checks connected to the corresponding variable node. In this paper, we analyze some typical error patterns where the variable node is erroneous, but the connected parity checks are satisfied (and vice versa). The proposed modification of the flipping rule takes into account these parity checks, that are no more assumed as valid indicators if the corresponding VN is correct. We have shown that the most significant performance improvement is obtained if the proposed modification is successively applied to the received word. We have also proposed a more general framework based on deterministic re-initializations of the decoder input in multiple decoding attempts.

The proposed algorithm results in significant performance improvement for the LDPC codes with various codeword lengths, obtained by using various construction procedures. It has been shown that the proposed algorithm can be extremely useful for short codes, even for a moderate number of iterations. When the maximum number of iterations can be increased, the proposed algorithm has the potential to provide very reliable transmission even for codes with large codeword lengths.

## Figures and Tables

**Table 1:** Computational complexity per decoding iteration.

Type of Operation	GDBF-w/m (Algorithm 1)	The Modification (Algorithm 2)
ρ-input XOR	*m*	*m*
(ρ−1)-input OR	-	<3m
1-bit comparison	-	<2n
Integer addition	2n	<5n
Global maximization	1	3
Integer comparison	*n*	<5n

## Data Availability

Not applicable.
